# Is There an Association Between Periodontitis and Gestational Diabetes? A Systematic Review and Meta-Analysis

**DOI:** 10.3390/dj14030139

**Published:** 2026-03-03

**Authors:** Ludovica Giancotti, Sara Sorrenti, Lorenzo Marini, Gregorio Volpe, Patrizia Gallenzi, Daniele Di Mascio, Andrea Pilloni, Antonella Polimeni, Giuseppe Rizzo, Umberto Romeo, Antonella Giancotti, Piero Papi

**Affiliations:** 1 Institute of Dental Clinic, A. Gemelli University Policlinic IRCCS, Catholic University of the Sacred Heart, 00168 Rome, Italy; 2 Department of Maternal and Child Health and Urological Sciences, Sapienza University of Rome, 00161 Rome, Italy; 3 Department of Oral and Maxillo-Facial Sciences, “Sapienza” University of Rome, 00161 Rome, Italy; 4 Clinic of General, Special Care, and Geriatric Dentistry, Center for Dental Medicine, University of Zurich, CH-8032 Zurich, Switzerland

**Keywords:** gestational diabetes mellitus, periodontitis, pregnancy, oral health, periodontal disease, hyperglycemia, meta-analysis, systematic review, maternal health

## Abstract

**Background:** Gestational diabetes mellitus (GDM) and periodontitis (PD) are chronic inflammatory conditions that may share metabolic and immune pathways. Evidence suggests an association between them, although results across studies remain inconsistent. This systematic review and meta-analysis evaluated the relationship between GDM and PD and examined whether GDM influences key periodontal parameters. **Methods:** A systematic search of PubMed/MEDLINE, Scopus, Web of Science, and Embase was conducted up to August 2025 following PRISMA guidelines. Observational studies comparing periodontal status in pregnant women with and without GDM were included. Periodontal status was assessed using probing pocket depth (PPD), clinical attachment loss (CAL), and bleeding on probing (BOP). Study quality was evaluated with the Newcastle–Ottawa Scale, and random-effects models were applied to estimate pooled associations. **Results:** Fifteen studies involving about 3800 pregnant women met the criteria. A significant association was found between GDM and PD (Odds Ratio [OR] 2.10; 95% Confidence Interval [CI] 1.64–2.69). No significant association emerged between GDM and gingivitis. Women with GDM showed increased BOP and higher PPD, indicating greater periodontal inflammation, while CAL did not significantly differ between groups. **Conclusions:** The findings support a significant association between GDM and periodontitis, suggesting that gestational hyperglycemia may enhance periodontal inflammation and early tissue changes. Incorporating periodontal screening into prenatal care may benefit maternal oral and metabolic health. Further longitudinal and interventional studies are needed to clarify causality and to explore whether periodontal therapy may help reduce risks linked to GDM.

## 1. Introduction

Periodontitis is a chronic inflammatory disease of the supporting tissues of the teeth, known as the periodontium, caused by a dysbiotic condition in which specific pathogenic bacteria, together with the host immune response, trigger progressive destruction of the periodontal ligament and alveolar bone [1]. This condition represents the main cause of tooth loss and, beyond its local effects, allows bacteria and inflammatory mediators to enter the systemic circulation, contributing to a state of chronic inflammation that may lead to major health complications [2]. Periodontitis (PD) is among the most common chronic diseases worldwide and has therefore been recognized as a significant global public health concern [3].

The relationship between PD and pregnancy outcomes has been increasingly investigated over the years [4,5]. Pregnancy is characterized by profound hormonal, microbiological, and immunological changes that can negatively affect oral health and exacerbate periodontal inflammation [6]. Conversely, the chronic inflammatory state associated with maternal PD may interfere with the course of pregnancy and contribute to adverse outcomes [7]. Evidence suggests that periodontal infection may act as a reservoir of bacteria capable of reaching the maternal–fetal unit through hematogenous dissemination [8]. Hence, Han et al. demonstrated the ability of certain periodontal pathogens, such as Campylobacter rectus (C. rectus), Fusobacterium nucleatum, and Porphyromonas gingivalis, to cross the placenta and cause an infection in the amniotic fluid [9]. Additionally, some observational studies have shown that pregnant women who underwent non-surgical treatment for PD showed reduced levels of inflammatory mediators in plasma, thus lowering the risk of adverse outcomes [10].

More specifically, gestational diabetes mellitus (GDM) refers to a glucose regulation disorder that is first diagnosed during pregnancy and typically develops during the second or third trimester. Although it generally resolves after delivery, GDM is associated with significant maternal and fetal morbidity [11]. From a pathophysiological perspective, pregnancy is a diabetogenic condition characterized by progressive insulin resistance, particularly from mid-gestation onward. This process is driven by hormonal changes related to the development of the fetal–placental unit, including the production of human placental lactogen, progesterone, cortisol, and prolactin, which interfere with insulin signaling [12,13].

It is believed that the source of pro-inflammatory cytokines in GDM may be the placenta and adipose tissue, though it is plausible that some of these molecules originate from inflamed periodontal tissues in pregnancies with PD, thus increasing the risk of developing GDM [14]. On the other hand, it has been observed that GDM and, thus, the hyperglycemic state, together with hormonal changes, exacerbate the periodontal inflammatory condition [15]. As a matter of fact, Abariga et al. [16], in their systematic review, concluded that PD is more commonly significant in women with GDM than in those without:; therefore, it can be hypothesized that there is a bidirectional association between these two pathological conditions, where the presence of one worsens the other, and conversely, treating one condition could lead to an improvement of the other. However, the association between GDM and PD is still controversial.

In a systematic review, Esteves Lima et al. state that, due to the clinical, methodological, and statistical heterogeneity of the studies evaluated, it is not possible to confirm a positive relationship between GDM and PD [17]. Non-significant results were also reported by other authors, who highlighted a rather homogeneous prevalence of periodontal disease between cases and controls [18,19].

Therefore, given the controversies in the literature, the objective of our study is to further explore the link between PD and GDM, in order to provide new public health intervention strategies for the prevention of GDM and its adverse effects on pregnancy outcome.

## 2. Materials and Methods

### 2.1. Protocol and Registration

This systematic review and meta-analysis were conducted at the Department of Oral and Maxillo-Facial Sciences, “Sapienza” University of Rome. The protocol was registered in the International Prospective Register of Systematic Reviews (PROSPERO), University of York, UK (registration number: CRD420251136701; available at: https://www.crd.york.ac.uk/PROSPERO/view/CRD420251136701 (accessed on 27 October 2025)). The study was performed in accordance with the Preferred Reporting Items for Systematic Reviews and Meta-Analyses (PRISMA) 2020 guidelines [20] (Supplementary File S1).

### 2.2. Focused Question and PECO Framework

The focused question guiding this review was:

“Do pregnant women with gestational diabetes have a greater prevalence and severity of periodontitis?”

This question was structured according to the PECO framework:

- Population (P): Pregnant women in the second and third trimesters;
- Exposure (E): Pregnant women diagnosed with GDM;
- Control (C): Pregnant women without a diagnosis of GDM;
- Outcome (O): Prevalence and severity of PD.

The primary outcome of this systematic review and meta-analysis was the prevalence of PD in pregnant women with GDM compared with normoglycemic pregnant women. Regarding periodontal disease severity, clinical attachment loss (CAL) was considered the primary parameter, as it reflects cumulative periodontal tissue destruction and is less influenced by transient inflammatory changes. Probing pocket depth (PPD) and bleeding on probing (BOP) were evaluated as secondary parameters indicative of current periodontal inflammation.

### 2.3. Search Strategy

An electronic literature search was performed in PubMed/MEDLINE, Scopus, Web of Science, and Embase from database inception up to 6 August 2025. The search strategy combined controlled vocabulary (MeSH terms) and free-text words related to gestational diabetes and periodontitis, using the Boolean operators “AND” and “OR.”

The complete search strategy was as follows:

(“Periodontal Diseases” [MeSH Terms]

OR “Chronic Periodontitis” [MeSH Terms]

OR “Aggressive Periodontitis” [MeSH Terms]

OR “Periodontitis” [MeSH Terms]

OR periodont* [All Fields])

AND

(“Diabetes, Gestational” [MeSH Terms]

OR “Pregnancy in Diabetics” [MeSH Terms]

OR “gestational diabetes” [All Fields]

OR (pregnancy [All Fields] AND diabetes [All Fields])

### 2.4. Eligibility Criteria

Studies were included if they met the following criteria:

- Design: Observational studies (cross-sectional, case–control, or cohort);
- Studies reporting the primary outcome were eligible for inclusion, regardless of whether secondary periodontal parameters were available;
- Studies conducted in human subjects;
- Articles published in the English language;
- No restrictions applied regarding year of publication.

Exclusion criteria included:

- Women with pre-existing type 1 or type 2 diabetes mellitus;
- Animal or in vitro studies;
- Reviews, case reports, editorials, and conference abstracts without full data;
- Studies with insufficient quantitative data for analysis.

### 2.5. Study Selection and Data Extraction

Two independent reviewers (LG and GV) screened all titles and abstracts for relevance, followed by full-text assessment of potentially eligible studies. Disagreements were resolved through discussion or consultation with a third reviewer (PP). The reference lists of all included papers and relevant reviews were manually screened to identify additional eligible studies.

Data extraction was performed independently using a standardized form that included:

- Study characteristics (authors, year, country, design, and sample size);
- Diagnostic criteria for GDM and PD;
- Participant characteristics (age, body mass index, gestational age, smoking status);
- Periodontal parameters and main findings.

### 2.6. Qualitative Assessment

The methodological quality of the included studies was independently assessed by two reviewers using the Newcastle–Ottawa Scale (NOS) [21]. For the qualitative evaluation, three different versions of the Newcastle–Ottawa Scale (NOS) were used, depending on whether the studies being assessed were cohort, case–control, or cross-sectional. This tool evaluates three domains: selection, comparability, and outcome/exposure. Studies were categorized as low, moderate, or high risk of bias according to their total NOS score.

### 2.7. Statistical Analysis

Before performing the meta-analysis, a descriptive analysis of the included studies was conducted. Study-level characteristics, including sample size, study design, geographic setting, diagnostic criteria for GDM and PD, and main periodontal outcomes, were summarized to provide an overview of the available evidence and to assess clinical and methodological comparability across studies. For continuous variables, means and standard deviations were reported, while frequencies and percentages were used for categorical variables, when available.

Based on this preliminary descriptive evaluation, a random-effects, head-to-head meta-analysis was conducted. This model was selected a priori to account for expected clinical and methodological heterogeneity among studies, including differences in study design, population characteristics, diagnostic criteria for GDM and PD, and periodontal assessment methods. For dichotomous variables, results were expressed as odds ratios (ORs) with 95% confidence intervals (CIs), and for continuous variables, as mean differences (MDs) with 95% CI. Heterogeneity was assessed using the I^2^ and τ^2^ (tau-squared) statistics. When more than 10 studies were available for a given outcome, funnel plots were constructed for exploratory assessment of publication bias.

When necessary, mean and standard deviation estimates were derived from median and range/interquartile values using the formula proposed by Wan et al. [22]. All statistical analyses were performed using Review Manager (RevMan), version 5.4.1 (Cochrane Collaboration, Oxford, UK).

## 3. Results

**Figure 1:**
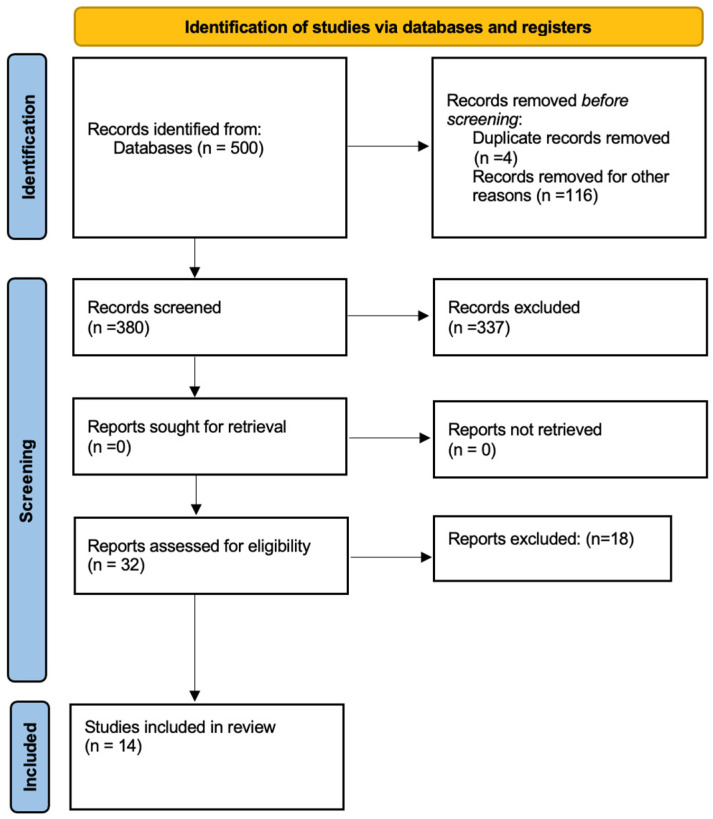
PRISMA flow diagram summarizing the study selection process, including records identified, duplicates removed, records screened, and studies included in the meta-analysis. n = number.

A total of 501 records were identified through database searching. After removal of duplicates, 380 studies were selected for title and abstract analyses, with 32 articles considered for detailed screening (Figure 1).

A total of 14 studies published between 2006 and 2024 were included in this systematic review, including case–control, cross-sectional, and cohort designs.

The included studies were conducted across 10 different countries, providing a broad geographical and ethnic representation. The main characteristics of the included studies are summarized in Table 1. The kappa agreement between reviewers was 0.85.

Overall, the pooled sample comprised approximately 3800 pregnant women, of whom around 980 were diagnosed with GDM, while 2820 served as non-GDM controls. Across most studies, participants were aged between 26 and 34 years. Women with GDM were generally older than normoglycemic controls, with a pooled mean age of 32.1 ± 4.6 years compared to 29.5 ± 4.7 years in controls, corresponding to an average difference of approximately 2.6 years (*p* < 0.001). Likewise, women with GDM consistently exhibited higher pre-pregnancy BMI values, ranging from 26.8 to 30.1 kg/m^2^, compared with 23.8 to 27.3 kg/m^2^ among controls (mean difference ≈+3.2 kg/m^2^, *p* < 0.001). The diagnosis of GDM was based predominantly on World Health Organization (WHO) or American Diabetes Association (ADA) criteria, applying standardized oral glucose tolerance tests (OGTT) [35,36].

Periodontal disease was identified through standard clinical parameters—Pocket Probing Depth (PPD), Clinical Attachment Loss (CAL), Bleeding on Probing (BOP).

While several studies adopted the 2017 World Workshop classification, other investigations relied on alternative diagnostic criteria, which may have contributed to variability in the assessment of periodontal outcomes [37,38,39].

### 3.1. Qualitative Analysis

According to the Newcastle–Ottawa Scale, the methodological quality of the included studies was overall moderate to high, with scores ranging from 6 to 7 out of 9 (Table 2). Most studies showed adequate case definition, appropriate control selection, and standardized periodontal assessment. Approximately two-thirds of the studies (8 out of 14) were rated as high quality (≥7 points), mainly those with larger sample sizes and adjustment for key confounders such as age, body mass index (BMI), and smoking. Lower scores were observed in older, single-center, or smaller studies, primarily due to limited comparability or restricted population representativeness. Complete assessment of the NOS can be found in Supplementary File S2.

Among the studies included in this systematic review, the majority reported evidence supporting a positive association between GDM and PD. Specifically, eight studies demonstrated statistically significant relationships, while the others did not reach statistical significance, although nearly all indicated a positive direction of effect. The strongest associations were observed in studies conducted by Cheng et al. [23], Şimşek et al. [24], Bunpeng et al. [25], and Damante et al. [26]. Specifically, Cheng et al. performed a large case–control investigation involving over 4800 women, reporting an odds ratio of 1.68 (95% CI 1.14–2.47; *p* < 0.001) and significantly higher mean PPD and CAL among women with GDM. Similarly, Şimşek et al. identified a markedly greater prevalence of PD in the GDM group (40%) compared with controls (21%; *p* = 0.006), emphasizing the inflammatory burden associated with metabolic dysregulation during pregnancy. Bunpeng et al. reported a comparable trend, with a twofold increased risk of PD among GDM patients (OR = 2.28; 95% CI 1.12–4.63; *p* = 0.021), while Damante et al. found one of the most pronounced prevalence differences (65% vs. 32%; *p* = 0.001). Additional studies conducted in diverse settings reinforced this association. Jamal et al. [27] observed an OR of approximately 1.7 (*p* = 0.02), Chokwiriyachit et al. [18] reported an OR of 3.0 (*p* = 0.02). Likewise, Xiong et al. [19] identified an OR of 2.5 (*p* = 0.014), further demonstrating the consistency of findings across populations and diagnostic criteria. Two studies obtained borderline results. Waligóra et al. reported a higher PD prevalence among women with GDM (23% vs. 10%; *p* = 0.06) [28] while Bullon et al. observed a similar trend (15% vs. 5%; *p* = 0.086) [29]. Conversely, a few studies—such as those by Esteves Lima et al. [30] Kumar et al. [31], Novak et al. [32], and Chaparro et al. [33]—did not detect significant associations.

### 3.2. Meta-Analysis

#### Periodontitis

**Figure 2:**
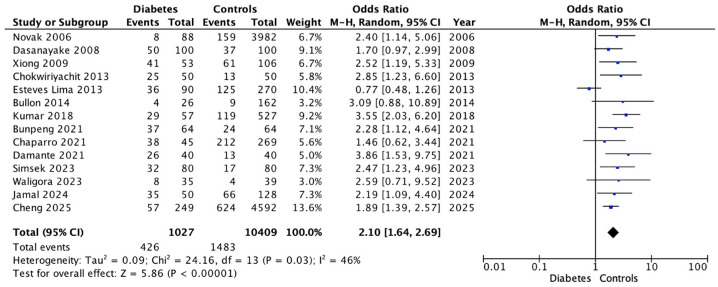
Forest Plot for Periodontitis showing the association between gestational diabetes and the outcome. Random-effects meta-analysis with odds ratios (OR) and 95% confidence intervals (CI). Studies included: Novak (2006) [32], Dasanayake (2008) [34], Xiong (2009) [19], Chokriyhachi (2013) [18], Esteves Lima (2013) [30], Bullon (2014) [29], Kumar (2018) [31], Bunpeng (2021) [25], Chaparro (2021) [33], Damante (2021) [26], Simsek (2023) [24], Waligora (2023) [28], Jamal (2024) [27], Cheng (2025) [23].

**Figure 3:**
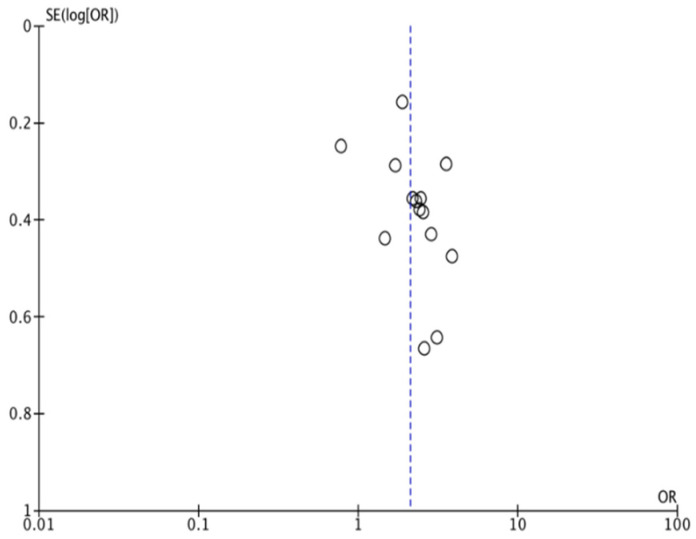
Funnel Plot Periodontitis.

When pooling all available data, the meta-analysis revealed that the presence of periodontal disease significantly increased the risk of developing GDM, with an overall odds ratio of 2.10 (95% CI: 1.64–2.69; *p* < 0.001). The heterogeneity index (I^2^) ranged from 35% to 45%, indicating moderate variability among studies (Figure 2 and Figure 3).

### 3.3. Gingivitis

**Figure 4:**
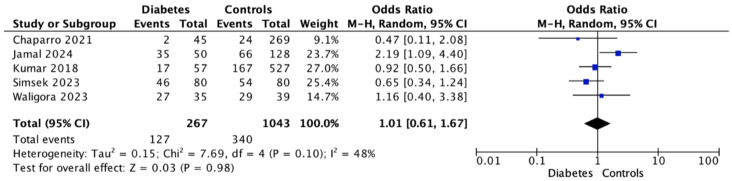
Forest Plot for Gingivitis showing the association between gestational diabetes and the outcome using a random-effects model. Odds ratios (OR) with 95% confidence intervals (CI) are presented. Studies included: Chaparro (2021) [33], Jamal (2024) [27], Kumar (2018) [31], Simsek (2023) [24], Waligora (2023) [28].

In contrast to the consistent association found between GDM and PD, the analysis did not reveal any significant link with gingivitis: based on the results of this meta-analysis, GDM is unlikely to influence the occurrence of gingival inflammation (OR = 1.01; 95% CI: 0.61–1.67; *p* = 0.98) (Figure 4).

### 3.4. Periodontal Parameters (BOP, PPD, CAL)

**Figure 5:**
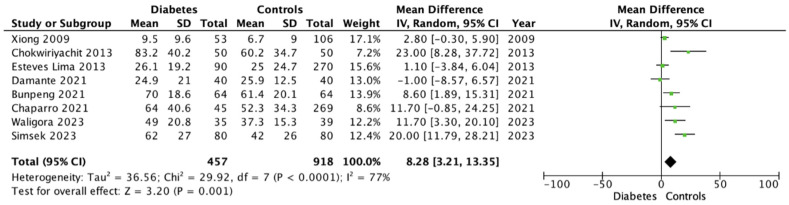
Forrest Plot for BOP showing mean differences between gestational diabetes and control groups using a random-effects model. Mean differences (MD) with 95% confidence intervals (CI) are presented. Studies included: Xiong (2009) [19], Chokwiriyachit (2013) [18], Esteves Lima (2013) [30], Damante (2021) [26], Bunpeng (2021) [25], Chaparro (2021) [33], Waligora (2023) [28], Simsek (2023) [24].

**Figure 6:**
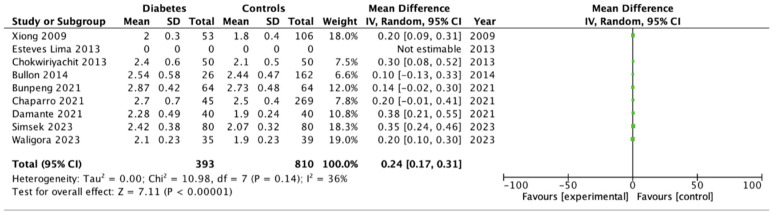
Forrest Plot for PPD mean differences between gestational diabetes and control groups using a random-effects model. Mean differences (MD) with 95% confidence intervals (CI) are presented. Studies included: Xiong (2009) [19], Esteves Lima (2013) [30], Chokwiriyachit (2013) [18], Bullon (2014 [29]), Bunpeng (2021) [25], Chaparro (2021) [33], Damante (2021) [26], Simsek (2023) [24], Waligora (2023) [28].

**Figure 7:**
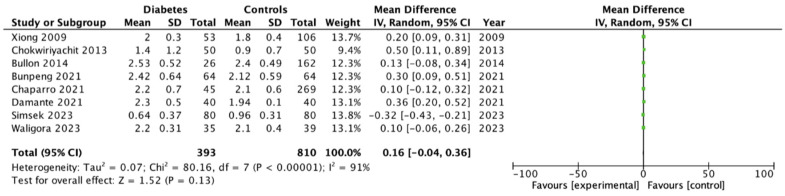
Forrest Plot CAL showing mean differences between gestational diabetes and control groups using a random-effects model. Mean differences (MD) with 95% confidence intervals (CI) are presented. Studies included: Xiong (2009) [19], Chokwiriyachit (2013) [18], Bullon (2014) [29], Bunpeng (2021) [25], Chaparro (2021) [33], Damante (2021) [26], Simsek (2023) [24], Waligora (2023) [28].

For continuous periodontal parameters, the pooled analysis indicated a consistent pattern of greater inflammatory and early periodontal changes among women with GDM. BOP was significantly higher in the GDM group, with a pooled mean difference of 8.3% (95% CI 3.2–13.4; *p* = 0.001), reflecting an enhanced gingival inflammatory response (Figure 5). PPD was also significantly increased (mean difference = 0.24 mm; 95% CI 0.17–0.31; *p* < 0.001) (Figure 6). In contrast, CAL showed no significant difference between groups (mean difference = 0.16 mm; 95% CI –0.04–0.36; *p* = 0.13) (Figure 7).

## 4. Discussion

Emerging evidence increasingly supports the concept that periodontitis induces a sustained inflammatory response that may extend beyond the oral cavity, influencing systemic immune and metabolic pathways [40]. This chronic low-grade inflammation could predispose pregnant women to develop GDM by aggravating insulin resistance, and the coexistence of these two conditions seems to intensify systemic inflammation and metabolic imbalance [6]. From a biological perspective, the association between GDM and PD may be explained by shared inflammatory and metabolic pathways. Both conditions are characterized by a state of chronic low-grade systemic inflammation, with increased circulating levels of inflammatory mediators such as C-reactive protein (CRP), interleukin-6 (IL-6), and tumor necrosis factor-α [41]. In pregnancies complicated by GDM, placental and adipose tissues contribute to an enhanced inflammatory response that promotes insulin resistance. Periodontal inflammation may act as an additional source of systemic inflammatory burden, further amplifying this response [42]. Moreover, elevated levels of matrix metalloproteinases (MMPs), which are involved in extracellular matrix degradation and tissue remodeling, have been reported in both PD and metabolic dysregulation, potentially contributing to periodontal tissue breakdown during pregnancy [28]. Although causality cannot be inferred from the available evidence, these shared inflammatory pathways provide a plausible mechanistic link between GDM and PD. Extending previous findings, our analysis aimed to further explore the relationship between GDM and periodontitis. What emerged from this systematic review and meta-analysis is a consistent and significant positive association between GDM and PD. The pooled analysis yielded an overall odds ratio of 2.10 (95% CI: 1.64–2.69; *p* < 0.001). These findings indicate that women with GDM are approximately twice as likely to present periodontitis compared with non-diabetic pregnant women. Before our meta-analysis, the study conducted by Estevens Lima et al. [17] was among the first to systematically investigate the potential association between PD and GDM: the authors included eight observational studies (five cross-sectional and three case–control) to determine whether PD increases the risk of developing GDM. Their findings indicated a significant association in cross-sectional studies, suggesting a stable and homogeneous relationship. In contrast, the association was not statistically significant in case–control studies, likely due to high heterogeneity and smaller sample sizes. In another systematic review, Abariga and Whitcomb [16] strengthened the existing evidence by including ten studies involving 5724 women and applying more rigorous methodological criteria. Through this comprehensive approach, the authors provided the most robust confirmation to date that PD nearly doubles the risk of developing GDM, even after adjusting for major confounding factors. The most recent meta-analysis by García-Martos et al. [43] confirmed previous findings by including eleven studies, showing that periodontal disease remains significantly associated with GDM despite moderate heterogeneity, even after controlling for BMI and socio-behavioral factors. Considering the cumulative evidence from previous meta-analyses supporting a potential link between PD and GDM, the present study was designed to further elucidate this relationship by reviewing the most recent studies published to date.

Taken together, the findings from the fifteen studies suggest that women with GDM tend to exhibit a higher prevalence of PD. Although statistical significance was not uniformly achieved, nearly all studies indicated the same positive direction of association. Significant associations were reported by Damante et al. [26], who observed a markedly higher prevalence of PD among women with GDM compared with controls (65% vs. 32%), and by Cheng et al. [23], a large case–control study involving more than 4800 participants, which found one of the strongest estimates (OR = 1.68; 95% CI). Similar findings were obtained by Şimşek et al. [32] reporting a significant association, and by Kumar et al. [16], who also noted a higher prevalence of PD among women with GDM. Collectively, these results reinforce the evidence that hyperglycemia-induced systemic inflammation may contribute to periodontal tissue breakdown [44]. In contrast, a smaller subset of studies reported non-significant findings. Waligora et al. and Esteves-Lima et al. presented borderline results, likely due to small sample sizes [28,30]. Chaparro et al. also found no difference between GDM and control groups [33]. Similarly, early investigations by Novak et al. and Dasanayake et al. did not achieve statistical significance, yet both reported a higher frequency of PD among women with GDM [32,34]. Although PD was the primary outcome of interest, gingivitis was retained as a secondary outcome to capture early inflammatory periodontal changes that may occur during pregnancy, particularly in the presence of metabolic alterations such as GDM. Unlike the clear association identified between GDM and PD, the evaluation did not uncover any meaningful connection with gingivitis; the findings imply that GDM is unlikely to play a role in the development of gum inflammation.

In addition to dichotomous outcomes, this meta-analysis also assessed continuous periodontal parameters—BOP, PPD, and CAL—to better characterize how GDM affects periodontal health. BOP, an indicator of superficial gingival inflammation, was significantly higher in women with GDM compared to controls, suggesting an exaggerated inflammatory response likely related to hyperglycemia-induced vascular changes and cytokine activation. PPD, reflecting active tissue breakdown, was also greater in the GDM group, indicating early periodontal destruction. In contrast, CAL did not differ significantly between groups, indicating that, in women with GDM, periodontal involvement is characterized by increased inflammatory activity and early periodontal changes, while no significant differences were observed in CAL values due to the short period of observation. These findings are highly consistent with the most recent meta-analysis by García-Martos et al., which reported almost identical mean differences for BOP and PPD and a similarly non-significant trend for CAL [43]. Both analyses confirm that GDM mainly amplifies gingival inflammation and shallow pocket formation, without yet producing clinically relevant attachment loss. In contrast, the earlier meta-analysis by Abariga and Whitcomb was limited to dichotomous outcomes and did not evaluate continuous indices such as BOP, PPD, or CAL [16]. The plaque index, despite being the primary etiological factor of periodontal disease, could not be included in the quantitative analysis because it was inconsistently reported and assessed using heterogeneous indices across the included studies, limiting comparability.

Several methodological aspects strengthen the present work. This review represents an updated synthesis of the literature and includes a relatively large number of observational studies conducted in different populations, resulting in an increased overall sample size. Furthermore, the methodological quality of the included studies, assessed using the Newcastle–Ottawa Scale, was generally moderate to high, supporting the reliability of the pooled analyses. At the same time, some limitations should be acknowledged when interpreting these findings. Most of the included studies had a cross-sectional design, which prevents any inference regarding the temporal or causal direction of the association between GDM and PD. Consequently, it cannot be determined whether periodontal disease contributes to the development of GDM or whether hyperglycemia and metabolic alterations associated with GDM negatively affect periodontal health. Also, variability in sample size across studies, particularly in smaller investigations with fewer than 100 participants per group, may have limited statistical power and contributed to heterogeneity in the reported results. An additional limitation is the heterogeneity of diagnostic criteria adopted across studies for GDM and PD, which may have introduced variability in case definitions and influenced the comparability of the findings.

## 5. Conclusions

This systematic review and meta-analysis identified an association between GDM and PD in pregnant women. Compared with normoglycemic controls, women with GDM showed a higher prevalence of PD. In addition, pooled analyses of continuous periodontal parameters indicated higher levels of BOP and greater PPD among women with GDM, whereas no statistically significant differences were observed in CAL. These findings were consistently reported across multiple studies, with differences observed across studies. Most of the included studies were rated as having moderate to high methodological quality, and standardized diagnostic criteria for both GDM and PD were commonly applied. Despite heterogeneity among studies and the predominance of cross-sectional designs, similar patterns of association were observed across different populations. Overall, the available evidence supports the presence of an association between GDM and PD as well as with specific periodontal clinical parameters, while highlighting the need for further well-designed longitudinal studies to better characterize this relationship.

## Figures and Tables

**Table 1:** Characteristics of the studies included in this systematic review.

REFERENCE	YEAR	STUDY DESIGN	COUNTRY	SAMPLE	AGE	BMI	DIAGNOSTIC CRITERIA PD	PD% IN GDM vs. CONTROL	PERIODONTAL INDICES GDM vs. CONTROL	G% GDM vs. CONTROL
Chokwiriyachit et al. [18]	2013	Case-control	Thailand	50 GDM/50 Control	33.5 ± 7.1 vs. 32.9 ± 7.1 *p* = 0.51	25.5 ± 4.8 vs. 22.4 ± 3.8 *p* = 0.001	≥1 site with both PPD ≥ 5 mm and CAL ≥ 2 mm	25/50 vs. 13/50 *p* = 0.02 OR 3.0	BOP(%) 83.2 ± 40.2 vs. 60.2 ± 34.7 *p* = 0.001; PPD (mm) 2.4 ± 0.6 vs. 2.1 ± 0.5 *p* = 0.02; CAL(mm) 1.4 ± 1.2 vs. 0.9 ± 0.7 *p* = 0.003	unknown
Xiong et al. [19]	2009	Case-control	United States	53 GDM/106 Control	29.9 ± 5.6 vs. 27.1 ± 5.99 *p* = 0.004	31.6 ± 8.1 vs. 25.7 ± 6.2 *p* = 0.000	≥1 site with PPD ≥4 mm or CAL ≥ 4 mm	41/53 vs. 61/106 *p* = 0.014 OR = 2.5	BOP (%) 9.5 ± 9.6 vs. 6.7 ± 9.0 *p* = 0.068; PPD (mm) 2.0 ± 0.3 vs. 1.8 ± 0.4 *p* = 0.028 CAL (mm) 2.0 ± 0.3 vs. 1.8 ± 0.4 *p* = 0.022	unknown
Cheng et al. [23]	2025	Case-control	China	249 GDM/4592 Control	unknown	unknown	Interdental CAL at ≥2 non adjacent or buccal CAL ≥ 3 mm with pocketing > 3 mm was detectable at ≥2 teeth	57/249 vs. 624/4592 *p* < 0.001 OR = 1.68 (IC:1.14–2.50)	unknown	unknown
Şimşek et al. [24]	2023	Cross-sectional	Turkey	80 GDM/80 Control	33.16 ± 5.16 vs. 29.33 ± 6.17 *p* = 0.066	32.43 ± 5.62 vs. 29.09 ± 5.44 *p* = 0.001	Interdental CAL at ≥2 non adjacent or buccal CAL ≥ 3 mm with pocketing > 3 mm was detectable at ≥2 teeth	32/80 vs. 17/80 *p* = 0.006	BOP (%) 62 ± 27 vs. 42± 26 *p* = 0.0001; PPD (mm) 2.42 ± 0.38 vs. 2.07 ± 0.32 *p* = 0.0001; CAL (mm) −0.64 ± 0.37 vs. −0.96 ± 0.31 *p* = 0.0001	46/80 vs. 54/80 *p* = 0.06
Bunpeng et al. [25]	2021	Cross-sectional	Thailand	64 GDM/64 Control	33.28 ± 5.20 vs. 31.09 ± 4.83 *p* = 0.015	24.89 ± 5.51 vs. 23.82 ± 5.03 *p* = 0.294	Interdental CAL at ≥2 non adjacent or buccal CAL ≥ 3 mm with pocketing > 3 mm was detectable at ≥2 teeth	37/64 vs. 24/64 *p* = 0.021 OR = 2.28 IC = 95% (1.12–4.64)	BOP (%) 70.03 ± 18.58 vs. 61.39 ± 20.10 *p* = 0.015 PPD(mm) 2.87 ± 0.42 vs. 2.73 ± 0.48 *p* = 0.038; CAL (mm) 2.42 ± 0.64 vs. 2.12 ± 0.59 *p* = 0.007	unknown
Damante et al. [26]	2021	Cross-sectional	Brazil	40 GDM/40 Control	32.5 (24.5–36.5) vs. 30 (27.5–33) *p* = 0.335	28.77 (24.12–32.95) vs. 24.19 (21.96–27.77) *p* = 0.002	Interdental CAL at ≥2 non adjacent or buccal CAL ≥3 mm with pocketing > 3 mm was detectable at ≥2 teeth	26/40 vs. 13/40 *p* = 0.001	BOP (%) 24.99 ± 21.04 vs. 25.99 ± 12.46 *p* = 0.796; PPD(mm) 2.28 ± 0.49 vs. 1.90 ± 0.24 *p* < 0.0001; CAL (mm) 2.27 (2.00–2.65) vs. 1.96 (1.86–2.0) *p* = 0.0001	unknown
Jamal et al. [27]	2024	Cross-sectional	Pakistan	50 GDM/128 Control	31.4 ± 5.2 vs. 29.2 ± 4.5 *p* = 0.01	unknown	≥4 teeth with ≥1 sites with PPD ≥ 4 mm and CAL ≥ 3 mm associated with BOP	35/50 vs. 66/128 *p* = 0.02 OR = 1.7 IC = 95% (0.95– 3.20)	unknown	35/50 vs. 66/128 *p* = 0.007
Waligora et al. [28]	2023	Case-control	Poland	35 GDM/39 Control	32 (29.0–34.0) vs. 30 (26.0–33.0) *p* = 0.390	23.8 (21.6–27.5) vs. 22.8 (21.8–23.6) *p* = 0.1700	Interdental CAL at ≥2 non adjacent or buccal CAL ≥3 mm with pocketing > 3 mm was detectable at ≥2 teeth	8/35 vs. 4/39 *p* = 0.060	BOP(%) 50 (35–62) vs. 36 (28–48) *p* = 0.020; PPD (mm) 2.1 (2.0–2.3) vs. 2.0 (1.8–2.1) *p* = 0.0002; CAL (mm) 2.2 (2.0–2.4) vs. 2.1 (1.8–2.3) *p* = 0.010	27/35 vs. 29/39
Bullon et al. [29]	2014	Cross-sectional	Spain	26 GDM/162 Control	32.4 ± 4.3 vs. 31.9 ± 4.41 *p* = 0.231	29.82 ± 6.11 vs. 26.94 ± 4.62 *p* = 0.440	≥2 interproximal sites with CAL ≥ 6 mm (not same tooth) and ≥1 interproximal site with PPD ≥ 5 mm	4/26 vs. 9/162 *p* = 0.086	BOP (mean n of sites) 0.36 ± 0.15 vs. 0.31 ± 0.22 *p* = 0.113; PPD(mm) 2.54 ± 0.58 vs. 2.44 ± 0.47 *p* = 0.418; CAL (mm) 2.53 ± 0.52 vs. 2.40 ± 0.49 *p* = 0.137	unknown
Esteves Lima et al. [30]	2014	Cross-sectional	Spain	26 GDM/162 Control	32.4 ± 4.3 vs. 31.9 ± 4.41 *p* = 0.231	29.82 ± 6.11 vs. 26.94 ± 4.62 *p* = 0.440	≥4 teeth with ≥1 sites with PPD ≥ 4 mm and CAL ≥ 3 mm associated with BOP	4/26 vs. 9/162 *p* = 0.086	BOP (mean n of sites) 0.36 ± 0.15 vs. 0.31 ± 0.22 *p* = 0.113; PPD(mm) 2.54 ± 0.58 vs. 2.44 ± 0.47 *p* = 0.418; CAL (mm) 2.53 ± 0.52 vs. 2.40 ± 0.49 *p* = 0.137	unknown
Kumar et al. [31]	2018	Cohort prospective	India	57 GDM/527 Control	unknown	unknown	≥4 teeth with ≥1 sites with PPD ≥ 4 mm and CAL ≥ 3 mm associated with BOP	29/57 vs. 119/527	unknown	17/57 vs. 167/527
Novak et al. [32]	2006	Cross-sectional	United States	88 GDM/3982 Control	unknown	Unknown	≥1 site with PPD ≥ 4 mm and CAL ≥ 2 mm, and BOP	8/88 vs. 159/3982 *p* = 0.2	unknown	unknown
Chaparro et al. [33]	2021	Cohort prospective	Chile	45 GDM/269 Control	30 (27–34) vs. 28 (25–32) *p* = 0.075	29.60 (26.75–32.45) vs. 27(24.42–31) *p* = 0.001	Interdental CAL at ≥2 non adjacent or buccal CAL ≥ 3 mm with pocketing > 3 mm was detectable at ≥2 teeth	38/45 vs. 212/269 *p* = 0.906	BOP (%) 69 (35–88) vs. 51(30–76) *p* = 0.082; PPD (mm) 2.8 (2.2–3.1) vs. 2.6 (2.2–2.8) *p* = 0.068; CAL (mm) 2.2 (1.8–2.7) vs. 2.0 (1.7–2.5) *p* = 0.244	2/45 vs. 24/269 *p* = 0.906
Dasanayake et al. [34]	2008	Cross-sectional	United States	100 GDM/100 Control	28.7 ± 5.3 vs. 26.6 ± 5.8 *p* = 0.07	30.5 ± 7.9 vs. 25.5 ± 4.4 *p*= 0.004	≥1 site with PPD ≥ 4 mm	50/100 vs. 37/100 *p* = 0.38	unknown	unknown

BOP Bleeding On Probing, BMI Body Mass Index, CAL Clinical Attachment Level, GDM Gestational Diabetes Mellitus, G Gingivitis, PD Periodontitis, PPD probing pocket depth,

**Table 2:** Newcastle–Ottawa Scale evaluation of studies included for qualitative analysis.

REFERENCE	SELECTION	COMPARABILITY	EXPOSURE	MAXIMUM POINTS
Chokwiriyachit et al. [18]	2	2	2	6
Xiong et al. [19]	3	2	2	7
Cheng et al. [23]	3	2	2	7
Şimşek et al. [24]	2	2	2	6
Bunpeng et al. [25]	3	2	2	7
Damante et al. [26]	3	2	2	7
Jamal et al. [27]	3	2	2	7
Waligora et al. [28]	2	2	2	6
Bullon et al. [29]	3	2	2	7
Esteves Lima et al. [30]	3	2	2	7
Kumar et al. [31]	2	2	2	6
Novak et al. [32]	3	2	1	7
Chaparro et al. [33]	2	2	2	6
Dasanayake et al. [34]	3	2	2	7

## Data Availability

The original contributions presented in this study are included in the article and supplementary material. Further inquiries can be directed to the corresponding author.

## Supplementary Materials

The following supporting information can be downloaded at: https://www.mdpi.com/article/10.3390/dj14030139/s1, Supplementary File S1: PRISMA 2020 checklist. Supplementary File S2: Complete evaluation of studies included using the Newcastle–Ottawa Scale.
