# Supplementary material for: Is There an Association Between Periodontitis and Gestational Diabetes? A Systematic Review and Meta-Analysis
Source: Dent J (Basel). 2026 Mar 3;14(3):139. doi: 10.3390/dj14030139 (PMC13025398; doi:10.3390/dj14030139)
Supplement: Supplementary file 1 [file dentistry-14-00139-s001.zip › dentistry-4098592-supplementaryS2.pdf]

Supplementary File 2 Newcastle–Ottawa Scale (NOS)

1. Case- Control Studies Qualification According to NOS.

Criterion	Waligora et al.	Cheng et al.	Xiong et al.	Chokwiriyachit et al.	Esteves Lima et al.
Selection					
1) Is the case definition adequate?					
a) Yes, with independent validation*	a*	a*	a*	a*	a*
b) Yes, by record linkage or based on self-reports					
c) No description					
2) Representativeness of the cases					
a) Consecutive or obviously representative series of cases*	b	a*	a*		a*
b) Potential for selection biases or not stated				b	
3) Selection of controls					
a) Community controls*					
b) Hospital controls	b	b	b	b	b
c) No description					
4) Definition of controls					
a) No history of disease (endpoint)*	a*	a*	a*	a*	a*
b) No description of source					
Comparability					
1) Comparability of cases and controls					
a) Study controls for maternal age*	a*	a*	a*	a*	a*
b) Study controls for BMI*	b*	b*	b*	b*	b*
Exposure					
1) Ascertainment of exposure					
a) Secure record (surgical records)*	a*	a*	a*	a*	a*
b) Structured interview masked to case/control status*					
c) Interview not masked					
d) Written self-report or medical record only					
e) No description					
2) Same method of ascertainment for cases and controls					
a) Yes*	a*	a*	a*	a*	a*
b) No					
3) Non-response rate					
a) Same rate for both groups*					
b) Non-respondents described					
c) Rate different and no designation	c	c	c	c	c
Maximum points	6	7	7	6	7

2. Cross Sectional Studies Qualification According to NOS.

Criterion	Bunpeng et al.	Damante et al.	Şimşek et al.	Jamal et al.	Bullon et al	Dasanayake et al	Novak et al
Selection							
1) Representativeness of the sample							
a) Truly representative*							
b) Somewhat representative*	b*	b*	b*	b*	b*	b*	b*
c) Selected group							
d) No description							
2) Sample size justification							
a) Justified and satisfactory*	a*	a*		a*	a*	a*	a*
b) Not justified			b				
3) Non-respondents							
a) Comparability between respondents and non-respondents established*							
b) No description of response rate	b	b	b	b	b	b	b
4) Ascertainment of exposure (risk factor)							
a) Validated measurement tool*	a*	a*	a*	a*	a*	a*	a*
b) Non-validated tool but described*							
c) No description							
Comparability							
1) Study controls for main factor*	a*	a*	a*	a*	a*	a*	a*
2) Study controls for additional factors*	b*	b*	b*	a*	b*	b*	b*
Outcome							
1) Assessment of outcome							
a) Independent blind assessment*	a*	a*	a*	a*	a*	a*	a*
b) Record linkage*							
c) Self-report							
d) No description							
2) Statistical test							
a) Test clearly described, appropriate, with CIs and p-value*	a*	a*	a*	a*	a*	a*	a*
b) Not appropriate, not described							
Maximum points	7	7	6	7	7	7	7

3. Cohort Studies Qualification According to NOS.

Criterion	Kumar et al.	Chaparro et al.
Selection		
1) Representativeness of the exposed subjects		
a) Truly representative*		
b) Somewhat representative*	b*	b*
c) Selected group		
d) No description		
2) Selection of the non exposed subjects		
a) Community controls		
b) Hospital controls	b	b
c) No description		
3) Ascertainment of exposure		
a) Secure record (surgical, clinical)*	a*	a*
b) Written self-report		
d) No description		
4) Demonstration that outcome was not present at start of study		
a) Yes*		
b) No	b	b
Comparability		
1) Comparability of cohorts on the basis of design or analysis		
a) Study controls for most important factor*	a*	a*
b) Study controls for additional factors*	b*	b*
Outcome		
1) Assessment of outcome		
a) Independent blind assessment*	a*	a*
b) Record linkage*		
c) Self-report		
d) No description		
2) Was follow-up long enough for outcomes to occur		
a) Yes*	a*	a*
b) No		
3) Adequacy of follow-up of cohorts		
a) Complete follow-up*		
b) Subjects lost to follow-up unlikely to introduce bias*		
c) Follow-up rate < adequate and no description	c	c
Maximum points	6	6
